# A comparison of machine learning models’ accuracy in predicting lower-limb joints’ kinematics, kinetics, and muscle forces from wearable sensors

**DOI:** 10.1038/s41598-023-31906-z

**Published:** 2023-03-28

**Authors:** Shima Mohammadi Moghadam, Ted Yeung, Julie Choisne

**Affiliations:** Auckland Bioengineering Institute, The University of Auckland, Auckland, New Zealand

**Keywords:** Biomedical engineering, Mechanical engineering

## Abstract

A combination of wearable sensors’ data and Machine Learning (ML) techniques has been used in many studies to predict specific joint angles and moments. The aim of this study was to compare the performance of four different non-linear regression ML models to estimate lower-limb joints’ kinematics, kinetics, and muscle forces using Inertial Measurement Units (IMUs) and electromyographys’ (EMGs) data. Seventeen healthy volunteers (9F, 28 ± 5 years) were asked to walk over-ground for a minimum of 16 trials. For each trial, marker trajectories and three force-plates data were recorded to calculate pelvis, hip, knee, and ankle kinematics and kinetics, and muscle forces (the targets), as well as 7 IMUs and 16 EMGs. The features from sensors’ data were extracted using the Tsfresh python package and fed into 4 ML models; Convolutional Neural Networks (CNN), Random Forest (RF), Support Vector Machine, and Multivariate Adaptive Regression Spline for targets’ prediction. The RF and CNN models outperformed the other ML models by providing lower prediction errors in all intended targets with a lower computational cost. This study suggested that a combination of wearable sensors’ data with an RF or a CNN model is a promising tool to overcome the limitations of traditional optical motion capture for 3D gait analysis.

## Introduction

Three-dimensional gait analysis (3DGA) provides quantitative information on the locomotion system and lower-limb functionality level during gait. 3DGA is an effective way to monitor changes in gait and is commonly used in hospitals and gait clinics. However, due to the optical motion capture (OMC) system cost and the time needed for pre- and post-processing of the data, gait clinics are sparse, and the waiting time to get assessed can become quite high. Moreover, OMC systems and force plates need to be set up in a controlled environment, such as a lab or a clinic, which has been shown to affect human gait^1,2^.

With the emergence of lightweight and inexpensive wearable sensors, collecting human gait data outside the clinic has been made feasible. Inertial Measurement Unit (IMU) and Electromyography (EMG) are two types of wearable sensors that are becoming widely used in 3DGA. IMUs are made of a single electronics module combining three accelerometers and three gyroscopes which respectively collect linear acceleration and angular velocity in 3 dimensions^3^. EMG electrodes are placed on the person’s skin at the muscle’s belly location and indirectly measure the electrical signals transmitted by motor neurons that cause muscles to contract^4,5^. Although wearable sensors are very promising in motion analysis, barriers exist to their widespread clinical adaptation. First, the integration of acceleration data to determine the IMUs’ position and orientation causes numerical drift errors over time^6^. Second, scaling the EMG signal to the patient’s maximum voluntary muscle contraction to determine muscle activation is not always feasible in patients with impaired muscle forces, such as stroke patients or children with Cerebral Palsy^7,8^.

To overcome challenges associated with wearable sensors’ data processing limitations, regression-based machine learning (ML) techniques can be used. ML models can establish a direct relationship between wearable sensors’ data and intended targets; such as joint kinematics, joint kinetics, and muscle forces in this study. Training an ML model would enable us to predict targets for either (1) a specific patient at a different time point/session (intra-subject model) or (2) additional unseen patients (inter-subject model). Furthermore, data-driven models will enable joint kinetics calculations without ground reaction force data (from force plates) and eliminate the need for expensive motion capture equipment. It is worth mentioning that for the intra-subject model, one session of data collection in a lab with an OMC system would be required to build a specific ML model for each participant. After one session of data collection in the lab (OMC + IMUs + EMGs), the IMU and EMG sensors’ data can be collected during rehabilitation and outside the clinic without the need for an OMC system to enable clinicians to quantify the patient’s recovery progress until the end of the treatment/procedure.

Neural Networks (NN), Random Forest (RF), and Support Vector Machines (SVM) are powerful ML algorithms that can be used for regression even when non-linearity exists inside the targets. In recent years, a few research groups have implemented these algorithms to estimate gait time series from wearable sensors. Some have looked at joint kinematics^9–22^, others at joint kinetics^12,23–25^ but also gait parameters such as stride length, velocity, and toe clearance^26–30^. To date, NNs are the most used ML model to predict joint kinematics and kinetics from IMUs^10,16,18,20,23,25,27,28,31,32^. Most of the mentioned studies^10,18,23,25,31,32^ used classic feedforward neural networks and achieved correlation coefficients higher than 0.86. However, it has been shown that convolutional neural networks (CNN) outperform classic NN models in gait time-series prediction, especially for joint kinematics^33,34^. To the best of our knowledge, there is only one research^35^ in which neural networks are implemented to predict muscle activations from EMG data to estimate joint kinetics in a forward dynamics model. Unfortunately, they did not determine muscle forces based on the predicted muscle activations. Bolam et al.^36^ developed an RF model to predict maximum knee flexion angle and provided a reliable workflow to remotely monitor post-operative progress in knee arthroplasty patients. Estimation of hip, knee, and ankle joint angles in the sagittal plane (but not joint kinetics and muscle forces) was performed in another study^17^ using five ML algorithms (multiple linear regression, RF, SVM, back propagation neural network and eXtreme gradient boosting). SVM models have been used mainly to predict gait parameters (stride length and width, stride time, and foot clearance)^26,29,30^ rather than the prediction of joint kinematics and kinetics. Multivariate Adaptive Regression Splines (MARS) is another powerful ML method that is an extension of linear models and automatically models non-linearities and interactions between variables. It seems that MARS has not been investigated to predict joint kinematics, kinetics, and muscle forces during 3D gait analysis yet.

Although the performance of different ML models has been investigated in some studies^10–19,23–32,35,36^, there is a lack of consensus on which ML algorithm is the most accurate for predicting joint kinematics, kinetics, and muscle forces. Most studies focused on specific joint angles or joint moments of the lower limbs in one plane. Furthermore, only a few studies used automatic feature extraction and selection to train each ML model^36–38^.

Therefore, the aim of this study was two-fold: (1) Extract features automatically and determine the most important features for the estimation of each target and (2) Compare the performance of four non-linear regression ML models (CNN, RF, SVM, and MARS) to estimate pelvis, hip, knee, and ankle joint angles, moments, and muscle forces in both intra-subject and inter-subject examinations. To this end, we employed a python package called Tsfresh^39^ to extract features from EMGs and IMUs data and developed each ML model by using the most important features. Finally, each ML model’s performance was evaluated based on its prediction accuracy and computational time.

## Methodology

**Figure 1:**
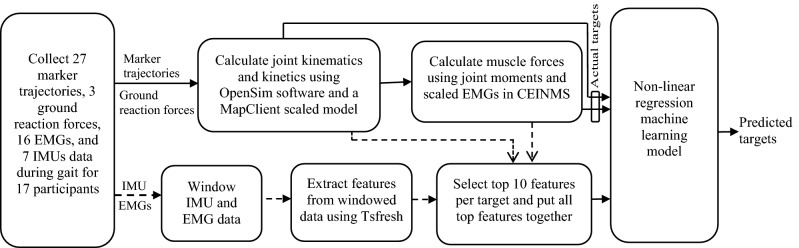
The workflow for developing ML models. The solid lines represent the process of generating desired outputs for ML models, and the dashed lines are related to the process of extracting and selecting features as models’ inputs.

The workflow to develop the ML models is represented in Fig. 1. The procedure of data collection, calculating targets from marker trajectories and ground reaction forces, extracting features from sensors’ data, and building ML models are explained in detail in the next paragraphs.

### Data collection

Seventeen healthy adults (9F, 28 ± 5 years, 1.70 ± 0.08 m, 66 ± 10 kg) with no recently reported injuries voluntarily participated in this study. Inclusion criteria were adults aged 18 years and older, and exclusion criteria were previous lower limb surgery, joint pain, osteoarthritis, or any other form of arthritis that would alter gait and any injury to the lower limbs in the past six months prior to the data collection. Each participant signed an informed consent form prior to collecting data in accordance with the World Medical Association Declaration of Helsinki (1964, last updated in 2013) and was approved by the University of Auckland (New Zealand) human participant ethics committee (reference number 019911).

**Figure 2:**
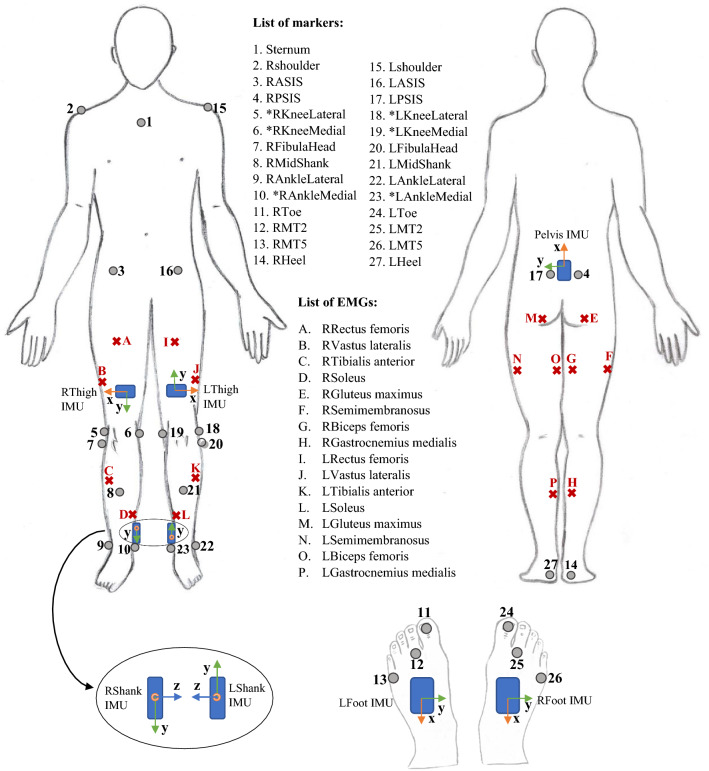
IMUs, EMGs, and OMC markers placement for data collection. The markers that their name starts with a * were only used for static trials and model scaling.

Participants were assessed in one session with at least one static, one squat, one squat jump, one heel raise, and sixteen over-ground walking trials with their self-selected speed. Each participant completed about ten gait cycles in each trial but only one gait cycle was used per trial in this study (the gait cycle that occurred over the force plates to calculate joint moments). The gait cycles used in the analysis were defined as the period of time from one heel strike of one foot to the next heel strike of the same foot. The steps in which the participant’s feet were outside the force plates were removed. After this initial data cleaning step, a different number of gait cycles remained for each participant (min = 8 and max = 24). The gait cycle duration for each participant varied based on their self-selected walking speed and step length. The minimum and maximum time for gait cycles were 0.75 and 1.25 s, respectively. In each trial, marker trajectories from a 12-camera optical motion capture system (Vicon Motion Systems Ltd., UK), ground reaction forces from three gound embedded force plates (Bertec, Columbus, Ohio), EMG (Mini-Wave, Italy), and IMUs (Vicon IMeasureU Ltd., NZ) were recorded. Twenty-seven reflective markers were placed on participants, as shown in Fig. 2, to determine the three-dimensional position and orientation of each body segment. Sixteen EMG surface electrodes were used to record lower limb muscles' activity on both legs (Gluteus maximus, Rectus femoris, Vastus lateralis, Biceps femoris, Semimembranosus, Medial gastrocnemius, Soleus, and Tibialis anterior). Three-dimensional acceleration and angular velocity were recorded from 7 IMUs attached to each segment of the lower limbs (one on the pelvis, one on each foot, shank, and thigh). With the exception of marker trajectories data, which was captured at a sampling frequency of 200 Hz, all data were recorded at 1 kHz. For each participant, maximum voluntary contraction (MVC) of lower limb muscles were also collected. During the MVC collections, the lab operator held the participant’s leg in a fixed position and asked them to move their leg with maximum effort to activate the group of muscles of interest. For each participant, the IMUs were taken off and repositioned in the middle of the session to account for the effect of small displacement in the IMUs’ position. Therefore half of the data captured was collected before and the other half after that extra step of removing and reattaching the IMUs on the participant’s skin. These trials were used randomly for training and testing for the intra-subject examination.

### Data post-processing

All captured data was synchronized, and marker trajectories were reconstructed through Vicon Nexus software (Version 2.12). The MOtoNMS^40^ (Matlab Motion data elaboration toolbox for neuromusculoskeletal applications) was used to filter 3D marker positions and force plate data (Butterworth 4th order, 10 Hz low pass filter) and rotate them according to the OpenSim coordinate system (X is perpendicular to the frontal plane pointing forward, Y is perpendicular to the transverse plane pointing upward, and Z is perpendicular to the sagittal plane pointing to the right). MOtoNMS was also used to process the EMG recording to determine muscles’ activations; (1) a zero-lag band-pass filter (4th order Butterworth 30–300 Hz), (2) full-wave rectification, and (3) a low pass filter (4th order Butterworth 4–10 Hz). Finally, the signals were normalized to the maximum value of EMG recorded during the MVC trials to scale underlying muscles' excitations as a number between 0 and 1.

An OpenSim model (gait2392)^41^ was scaled using the MAP-client workflow^42^ based on marker data using Principal Component Analysis to build a personalized musculoskeletal model for each participant. Pelvis, hips, knees, and ankles kinematics and kinetics were computed using the OpenSim inverse kinematics (IK) and inverse dynamics (ID) tools, respectively (version 3.3). The Calibrated EMG-Informed Neuromusculoskeletal Modelling (CEINMS)^43^ toolbox was used to estimate muscle forces. To calibrate musculotendon units (MTUs), we used three walking, one heel raising, and one squat trial in the CEINMS calibration step to adjust musculotendon parameters like tendon slack length, optimal fiber length, and strength coefficient. The objective function for calibration was defined by minimizing the differences between the joint moments estimated by the EMG-driven model and those derived from inverse dynamics during multiple calibration trials. Once calibration was completed, all MTUs activation and forces were predicted using the EMG-assisted approach (hybrid mode) of CEINMS. Finally, IMU, EMG, joint angles, joint moments, and muscle forces data were down-sampled to 100 Hz to decrease the computational cost of feature extraction and ML models construction. Joint moments were normalized to each participant’s body weight.

### Models’ development

The development process of each ML model to predict the targets (joint angles, joint moments, and muscle forces) from wearable sensors’ data is explained below. To increase the predictive power and facilitate the ML process, we identified all features from raw IMU (acceleration and angular velocity in three directions) and EMG data by using an open-source python package called Tsfresh (Time Series FeatuRe Extraction on basis of Scalable Hypothesis tests)^39^. In order to prepare the IMU and EMG data for Tsfresh, we put them into sequences of consecutive and overlapping windows, where a window is shifted across the data points to create smaller segments of time series signals per target value. In this study, the window size was one second, as recommended by Banos et al.^44^.

#### Feature extraction and selection

The minimum and maximum number of gait cycles for participants were 8 and 24, respectively. The length of gait cycles was also different (between 0.75 and 1.25 s) based on participants’ self-selected walking speed and step length, as mentioned in 2.1. However, the total number of data points was 37,579 for all participants. Each participant’s data was split into training (70% of gait cycles) and testing (the remaining 30% of gait cycles) sets. All participants’ training data (26,298 data points) were used for feature extraction and selection procedure. Tsfresh extracted 788 features from each channel of IMU and EMG data. A total of 58 channels were available from all IMUs and EMGs; 42 channels from seven IMUs (each IMU had six components: triaxial gyroscope and triaxial acceleration data), and 16 channels of EMGs. From these 58 channels, 45,704 features were extracted. In order to increase the prediction power of ML models, we eliminated irrelevant features that were not providing information to predict our targets. Removing unnecessary features will also decrease computational cost and time, which is crucial for real-world applications. First, for each target, a primary selection step took place to retain features with non-zero variance (31,487 features remained). Then extra feature selection procedures were performed to find the most important features related to each target by removing non-significant features in predicting target values. Then, the rank of each feature was determined based on Gini Importance^45^ for predicting each target using an RF regressor. Finally, the top ten features related to each target were selected. All top features were put together to build a super feature set, including 500 features (10 for each of the 50 targets). The final feature set was further reduced by removing repeated features to avoid redundancy, resulting in a total of 441 features.

#### Non-linear regression ML models

The most important features extracted by Tsfresh were used as inputs to the ML models (CNN, RF, SVM, and MARS) to predict the targets (joint kinematics, joint kinetics, and muscle forces) in over-ground walking. All ML models were multi-output, which predicted all targets simultaneously. Scikit-learn (a python library for ML) was used to set up the CNN, RF, and SVM models, while the MARS model was built using the py-earth python library. To optimize the performance of the models, the hyperparameters were tuned using the following approach. The data from all participants were divided into two sets: a training and validation set (80% of the data) and a testing set (20% of the data). We used a five-fold cross-validation on the first set to determine a combination of parameters that resulted in the lowest error. It involved splitting the data into five equally sized subsets, or "folds." The model was then trained on four of the folds and evaluated on the fifth fold. This process is repeated five times, with each fold being used as the evaluation set once. Finally, the testing set was used to evaluate the final performance of the model. To perform the hyperparameter search, the GridSearchCV method was utilized, which searches over the hyperparameters defined in the parameter grid. This approach ensures that the model's hyperparameters are optimized while minimizing the risk of overfitting, ultimately leading to a robust and accurate model. The tunned hyperparameters found for each ML are described below.

CNNs are a specialized type of NN model that has shown remarkable performance in various tasks, including gait time-series prediction. This study used a multi-output CNN model with five hidden layers to estimate joint kinematics, joint kinetics, and muscle forces. First, we used the StandardScaler function from the sklearn library for scaling features to ensure all variables are in the same range (between zero and one). It was also necessary to scale targets as we used a multi-output CNN model. Targets were scaled back to their original scale using the same scaler after predictions. Then, the model's architecture was defined with an input layer size of 441. Then two convolutional layers were added, each followed by a max pooling layer. Both convolutional layers had 256 filters with a kernel size of three and a “relu” activation function. The max-pooling layers had a pool size of two. These layers helped reduce the data's dimensionality and identify the most prominent features of the previous feature map. After the max-pooling layers, the data was flattened and passed through the output layer, which was a dense layer with a linear activation function. The number of units in the output layer was equal to the number of targets (50). The 'Adam' solver (with a learning rate of 0.01), a stochastic gradient-based optimizer, was used for weight optimization, and the loss function was “mean squared error”. The EarlyStopping function was used to monitor the validation loss and stop the training if the loss did not improve after five epochs. The batch size was set to 32, and the model was trained for a maximum of 100 epochs. Supplementary Fig. 1 represents the loss versus the number of epochs for training and validation. In this model, the optimal activation function (among 'relu', 'sigmoid', and 'tanh') in hidden layers, the optimizer (among 'adam', 'rmsprop', and 'sgd') and its learning rate (among ‘0.1’, ‘0.01’, and ‘0.001’), and the number of neurons (among 64, 128, and 256) in each convolutional layer were found through grid search.

RF is a flexible and easy to use ML model for regression. RF builds forest (ensemble of decision trees) trained with the bootstrap aggregating (bagging) method and outputs the average of prediction of individual decision trees^46^. The RF model's tunned hyperparameters in this study were the number of trees (among: 100, 200, 300, 400, and 500), the maximum number of randomly selected variables in each tree (among 'auto', 'sqrt', and 'log2'), and the maximum depth of each tree (among 15, 20, 25, and 30). Based on the grid search, the final combination of hyperparameters that provided the lowest error was as follows: 500 for the number of trees (Increasing the number of trees can improve the model's performance, but it may also increase the computational complexity and training time which might not be ideal for using the model in real applications), ‘sqrt’ for the number of randomly selected features which means the root square of the number of inputs (in this study, this number was equal to 21 as the number of input variables was 441), and the maximum depth of 25 for each tree.

Another powerful supervised learning model for non-linear regression is SVM^47^. In this model, a threshold (ε) is set by the user to control the maximum allowable error for the regression setting. When there is non-linearity in the dataset, a kernel function is used to map the input feature vectors to a higher dimensional feature space. As SVMs are sensitive to the scale of features, we performed feature scaling to improve this model’s performance. In this study, after hyperparameters tunning, we set $\varepsilon =$ 0.01 (among 0.001, 0.01, 0.1, and 1), cost parameter $C=$ 10 (among 0.1, 1, 10, and 100), and radial basis function ‘rbf’ as the kernel function (among 'linear', 'rbf', and 'sigmoid').

The last model developed in this study was a MARS which is well suited for high-dimensional problems. MARS is an extension of linear models by modeling non-linearities in target values. This model aggregates a set of simple linear functions' results to perform well in predicting any kind of target vector. MARS algorithm automatically discovers the number and type of basis functions to use. We set the number of input variables considered by each piecewise linear function (max_degree) to two (among 1, 2, and 3). The maximum number of basis functions (max_term) was 100 (among 100, 200, and 300).

### Performance evaluation

#### Intra-subject examination

To investigate each ML model’s performance for predicting the targets for the same participant, the intra-subject examination was performed. In the intra-subject examination, we used 70% of one participant’s gait data to train the ML model, and we tested the model on the remaining 30% of the same participant’s data. This examination was done for all participants (creating 17 different models, 1 per participant).

#### Inter-subject examination

The inter-subject examination evaluated the ML models’ performance to predict targets for an unseen participant. Leave-one-out (LOO) cross-validation was performed to investigate ML models’ generalizability. A LOO analysis consists of splitting the training (N-1 participants) and testing (1 participant) dataset N times, with N = number of participants. Therefore we created 17 training/testing combinations of partcipants data to build 17 ML models. Each time 16 participants’ data were used for training the ML model, and the model was tested on the remaining participant’s data.

#### Performance metrics

To compare the performance of the ML models in the testing datasets, the Root Mean Square Error (RMSE), Mean Absolute Error (MAE), and coefficient of determination (${R}^{2}$) between the computed and predicted targets were calculated for each gait cycle and each participant for both intra and inter-subject examinations. In order to make a better interpretation of muscle forces errors for each muscle, we reported NRMSE (RMSE normalized to the range of data). The reported RMSEs, NRMSEs, and MAEs are the average of cross-validation for all participants. The ${R}^{2}$ values are presented in percentages and calculated for each target using predicted data from all participants to have a single value.

To identify the most computationally efficient model and investigate the effect of feature selection on the ML model’s performance, an additional examination was performed. All models were trained (on 16 randomly selected participants’ data) and tested (on the remaining participant’s data) twice. The first time, we used all features with non-zero variance (31,487 features), while the second time, we only utilized the selected features (441 features). For both cases, the prediction accuracy and computational time were recorded. This examination enabled us to identify the model that can be effectively applied in a practical setting across various systems and clinics.

## Results

The most important features selected to predict each target are available in the Supplementary Table S1. The top features were extracted from the IMU signals for kinematics and kinetics prediction. In contrast, most of the top features were extracted from EMG signals for muscle forces prediction. According to Supplementary Table S2, the highest and lowest computational times for testing the models were related to SVM and CNN models respectively. The testing time for the CNN and RF models using only the selected features (441 features) took less than a second, while the testing time for the SVM was 945 s. Using all non-zero variance features (31,487 features) to train and test the models increased computational time, especially for SVM and MARS models. While the prediction accuracy improved slightly for the CNN model, the performance of other models worsened by using all non-zero variance features instead of the selected features (Supplementary Table S3).

The following results represent the performance of each ML model, CNN (pink), RF (blue), SVM (yellow), and MARS (green), for the prediction of intended targets (joint kinematics, joint kinetics, and muscle forces).

### Joint kinematics

**Figure 3:**
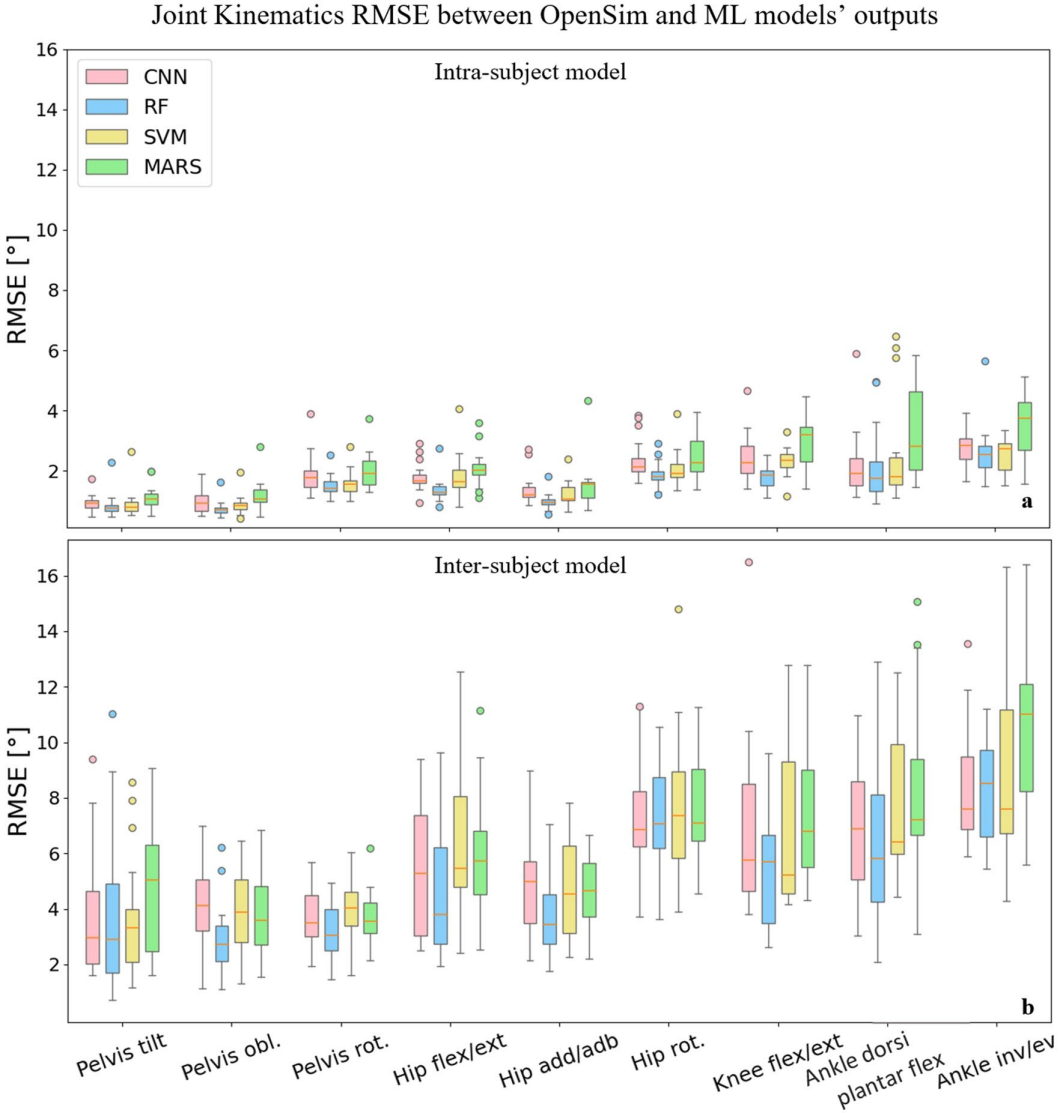
RMSE values across joints and planes of motion between OpenSim inverse kinematics and ML models’ joint angles predictions in intra-subject (**a**) and inter-subject (**b**) examinations for all participants.

Although CNN, RF, and SVM models' performance were in the same range for most joint angles, the RF model provided the lowest RMSEs in all joints and planes of motion compared to other models for the intra-subject examinations (Fig. 3). The most accurate estimations for the inter-subject examination were provided by the RF and CNN models. The CNN model outputted the lowest prediction errors in pelvic tilt, hip rotation, and ankle inversion/eversion angles. The best performance for knee flexion/extension angle was related to the SVM model, and for the rest of the joint angles, RF provided the lowest error. The highest RMSE was related to MARS predictions in most joint angles in both intra and inter-subject examinations. The lowest joint angles RMSE in the RF model were related to pelvic obliquity (RMSE = $0.74^\circ$ for intra-subject examination and $2.95^\circ$ for inter-subject examinations), and the highest error was found for ankle inversion/eversion (RMSE = $2.58^\circ$ for intra-subject examination and $8.32^\circ$ for inter-subject examinations). The same trend can be seen by investigating other evaluation metrics, such as MAE and the ${R}^{2}$ values presented in the Supplementary Table S4.

**Figure 4:**
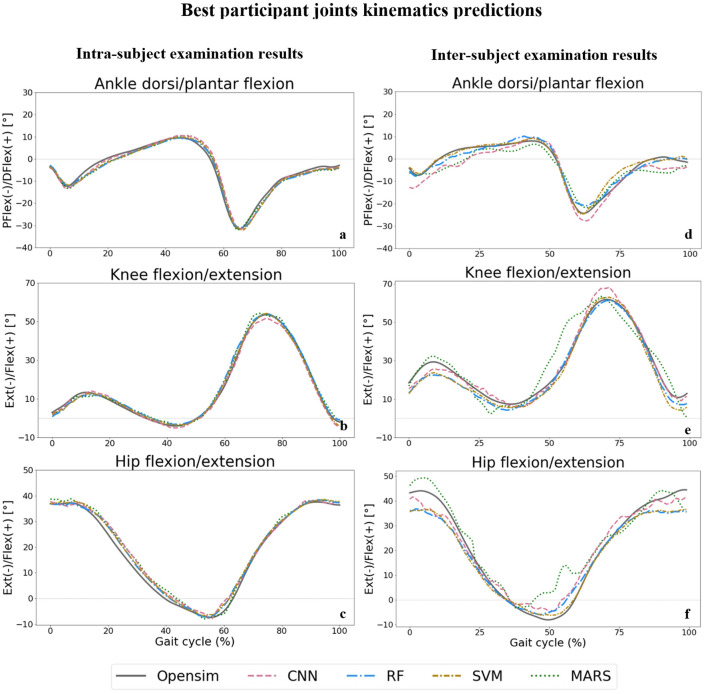
Joint angles predictions by ML models compared to joint angles derived from OpenSim IK tool (solid grey line) across one gait cycle for ankle dorsi/plantar flexion (**a** for intra and **d** for inter-subject), knee flexion/extension (**b** for intra and **e** for inter-subject), and hip flexion/extension (**c** for intra and **f** for inter-subject) angles.

Examples of the hip, knee, and ankle sagittal plane ROM during a gait cycle for the best participants (based on RF results) for the intra and inter-subject examinations are displayed in Fig. 4. The worst participant's results and other joint kinematics are shown in Supplementary Figs. S2 and S3 for one gait cycle. As can be seen, the RF model (dashed blue line) provided the best predictions by following OpenSim inverse kinematics output (solid grey line) better than other models.

### Joint kinetics

**Figure 5:**
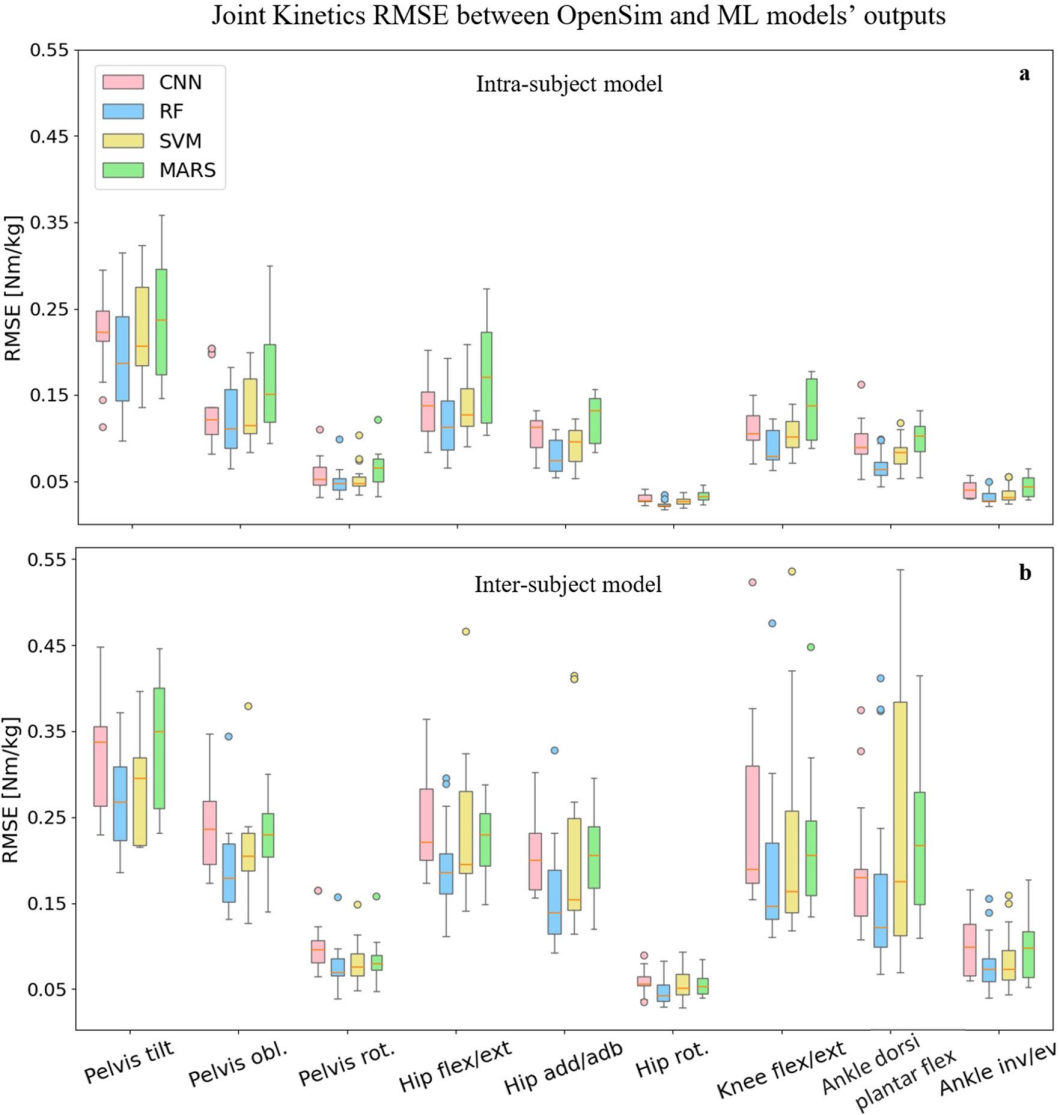
RMSE values across joints and planes of motion between OpenSim inverse dynamics and ML models’ joint moments' predictions in intra-subject (**a**) and inter-subject (**b**) examinations for all participants.

The RF model consistently provided lower RMSE than other ML models in all joints moments’ predictions, analogously to the kinematics results (Fig. 5). The MARS model produced the maximum RMSE in most joints for the intra-subject examinations, with the worst predictions provided the by SVM and MARS models for the inter-subject examination. RF model’s RMSE in joint kinetics prediction ranged between 0.023 Nm/kg (hip rotation moment) and 0.191 Nm/kg (pelvic tilt moment) in the intra-subject examination. The minimum and maximum joint kinetics RMSE were 0.047 Nm/kg (hip rotation moment) and 0.269 Nm/kg (pelvic tilt moment) in the inter-subject examination for the RF model. MAE and ${R}^{2}$ values are presented in Supplementary Table S5 for joint kinetics predictions by all models.

**Figure 6:**
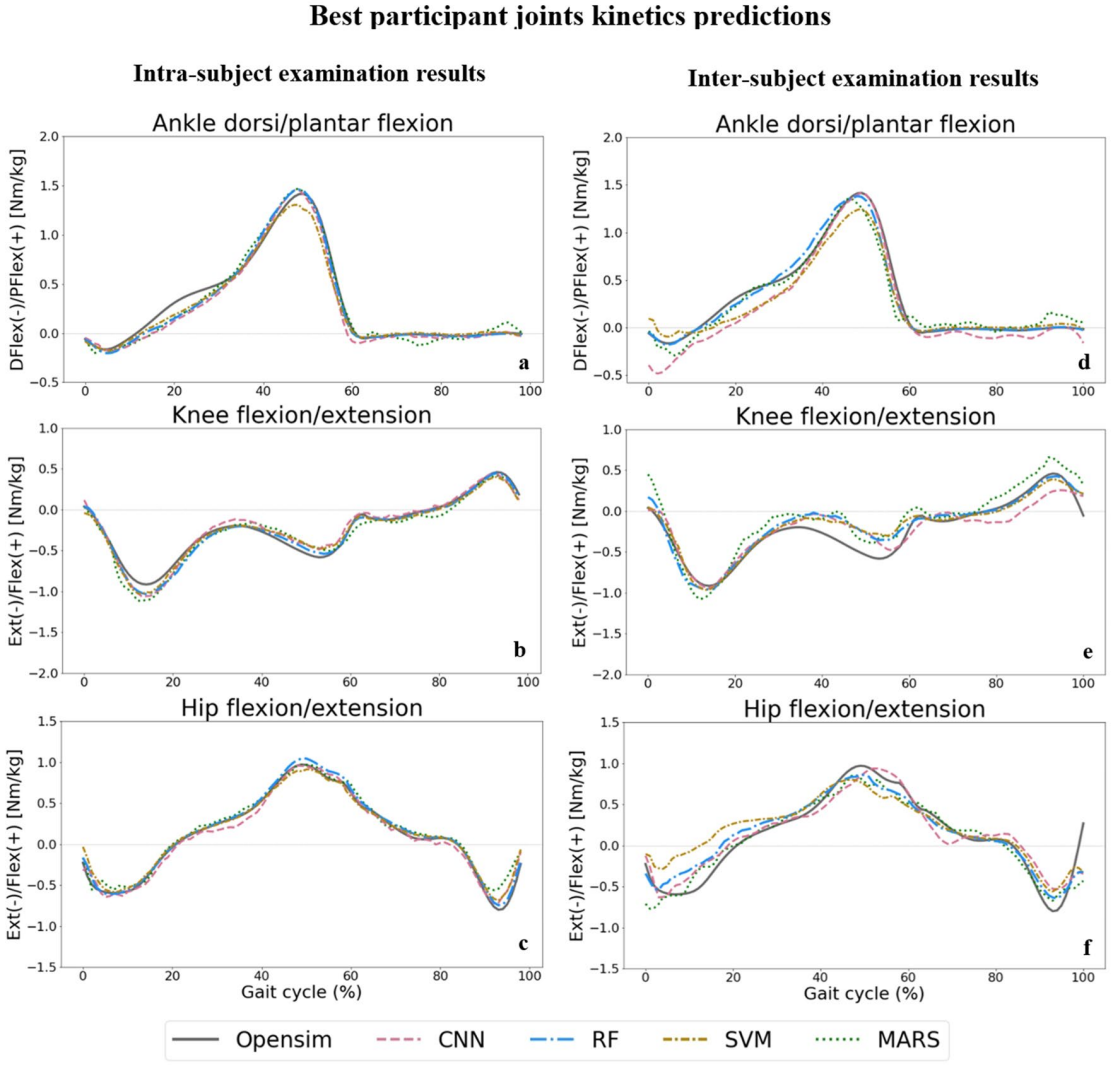
Joint moments predictions by ML models compared to joint moments derived from OpenSim ID tool (solid grey line) across one gait cycle for ankle dorsi/plantar flexion (**a** for intra and **d** for inter-subject), knee flexion/extension (**b** for intra and **e** for inter-subject), and hip flexion/extension (**c** for intra and **f** for inter-subject) moments.

The best participant predictions for all ML models for one gait cycle for ankle, knee, and hip moments in the sagittal plane are displayed in Fig. 6. The worst participant predictions and other joints kinetics are presented in Supplementary Figs. S4 and S5 for a gait cycle. The RF model (dashed blue line) provided better predictions of the OpenSim inverse dynamics outputs (solid grey line) compared to the other models.

### Muscle forces

**Figure 7:**
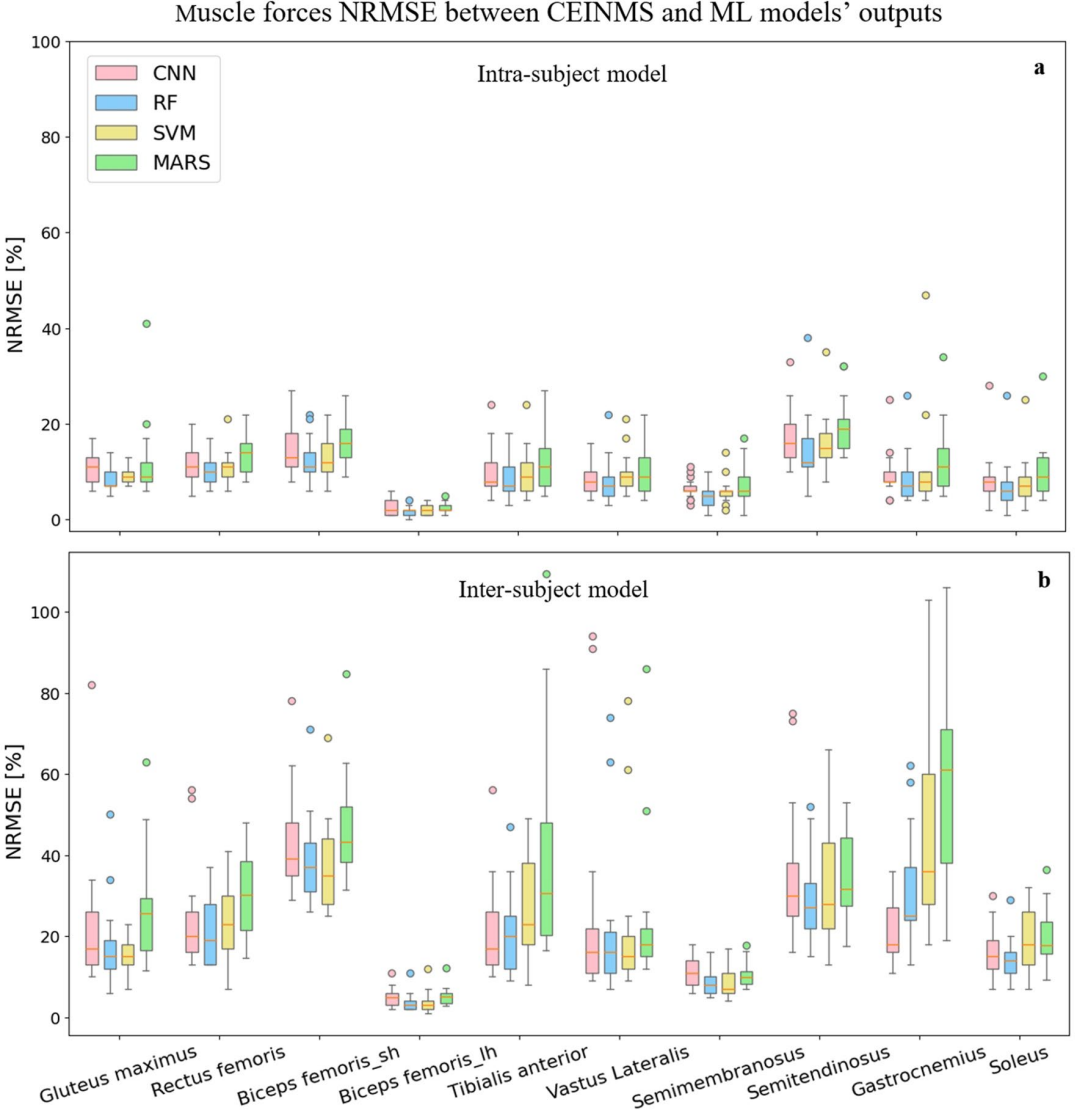
RMSE values between CEINMS muscle forces and ML models’ predictions in intra-subject (**a**) and inter-subject (**b**) examinations for all participants. Biceps femoris short heat and biceps femoris long head muscles are shown as Biceps femoris_sh and Biceps femoris_lh, respectively.

To predict muscle forces, the RF and CNN models displayed the lowest NRMSE between CEINMS outputs and prediction output from ML models, while the highest NRMSEs came from the MARS model (Fig. 7). In the inter-subject examination, the CNN model outperformed the RF model for the tibialis anterior and gastrocnemius muscles prediction. The maximum average NRMSE value occurred when predicting the semitendinosus muscle force (NRMSE of 14.1%) for the intra-subject examination and biceps femoris short head muscle (NRMSE of 36.2%) in the inter-subject examination. The average RMSE for the biceps femoris long head muscle force prediction was the lowest among all muscles for all ML models in both intra-subject (NRMSE of 2.6%) and inter-subject (NRMSE of 4.5%) examinations. MAE and ${R}^{2}$ values between models’ predictions and CEINMS output are presented in Supplementary Table S6 for the muscle forces predictions.

**Figure 8:**
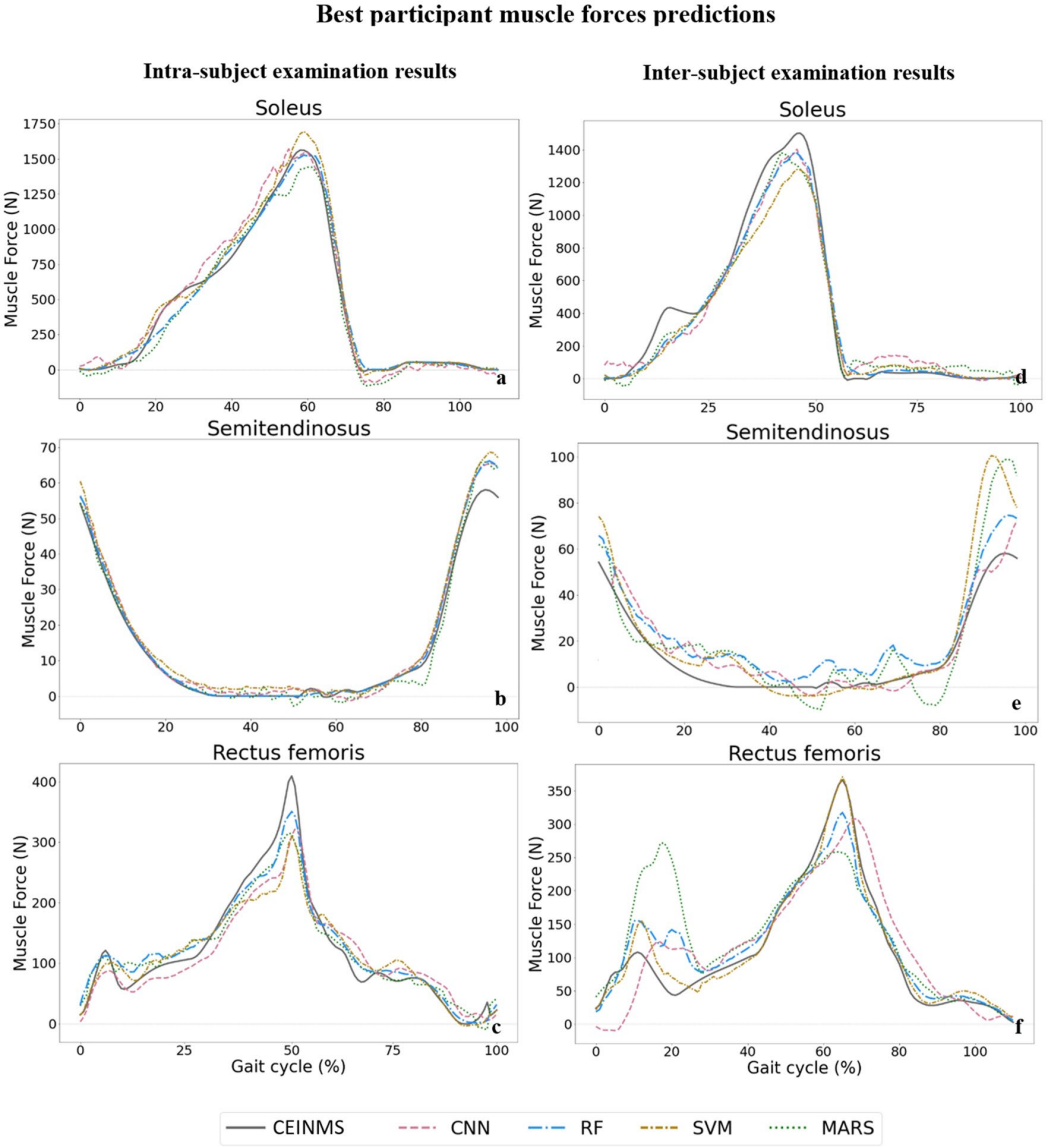
Muscle forces predictions by ML models compared to CEINMS outputs (solid grey line) across one gait cycle for soleus (**a** for intra and **d** for inter-subject), semitendinosus (**b** for intra and **e** for inter-subject), and rectus femoris (**c** for intra and **f** for inter-subject).

Muscle forces predictions by all models can be seen in Fig. 8 for the soleus (ankle plantar flexor muscle), semitendinosus (knee flexor muscle), and rectus femoris (hip flexor muscle) across one gait cycle for the best participants in each examination. Figures demonstrating the worst participant's results and other muscle forces are available in Supplementary Figs. S6 and S7. The best match between the ML models’ prediction and CEINMS output came from the RF model for both intra-subject and inter-subject examinations.

## Discussion

This study aimed to compare the performance of ML models for the prediction of important lower-limb gait time series (joint kinematics, joint kinetics, and muscle forces) from wearable sensors’ data with the aid of automatic feature extraction. The first objective of this study was to extract features automatically and determine the most important features for the estimation of each target. To extract all possible features from raw EMG and IMU data, we used a python package called Tsfresh^39^. Tsfresh’s ability to extract a high number of features and determine their significance makes it more suitable than manual feature extraction methods. Furthermore, the most important features that are essential for predicting a particular target might be neglected when extracted manually^48^. As a result, the top features to predict joint kinematics and kinetics were extracted from the IMU data, and the top features for most of the muscle forces were extracted from EMG data. These results were predictable, as the joint angles are closely related to the angular velocity (gyroscope data), joint moments are associated with linear acceleration (accelerometers data) and angular velocity, and EMG data are correlated with muscle activation and, therefore, muscle forces. Interestingly, the most important features for some targets appeared unrelated. For example, we found that a feature extracted from the z-axis of gyro data from the thigh sensor was the most informative feature for predicting hip flexion/extension angle and hip abduction/adduction. It may suggest that the sensors' axes may not be perfectly aligned with the joint axes of rotation in the body.

Despite the parallelization of the extraction and selection tools in tsfresh, the memory consumption of parallel calculations can be high. Tasks with a high number of processes may be limited to machines with low memory. Therefore, reducing the number of features enable the use of our workflow on any system. Although feature selection can extend the time required to train models, it can significantly reduce the time needed for model’s inference. Our experiments showed that including all non-zero variance features could increase testing time for all models. This is especially important for real-world clinical applications, where efficient models with lower computational costs are essential. However, our findings also suggest that including all non-zero variance features does not necessarily improve model performance. In fact, using all features can actually worsen the performance of the RF, SVM, and MARS models by including many unrelated features. While the use of all non-zero variance features slightly improved the performance of the CNN model, the improvements were not substantial. Therefore, careful feature selection is important for developing accurate and efficient models. The second objective of this study was to compare the performance of four non-linear regression ML models (CNN, SVM, RF, and MARS) to estimate pelvis, hip, knee, and ankle joint angles, moments, and muscle forces in both intra-subject and inter-subject examinations. The ML models’ performance were compared based on their resulting RMSE, MAE, and R^2^ against the OpenSim and CEINMS output (used here as ground truth). The computed OpenSim joint angles and moments waveforms found in this study were similar to the literature^49,50^. Muscle forces computation were validated by comparing them to the experimental EMG recordings.

We found that the RF and CNN models performed best in predicting joint kinematics and muscle forces for the intra and inter-subject examinations, as they provided the lowest prediction errors and computational time to be trained and tested. The SVM model also provided prediction errors in the range of the RF and CNN models in some joint angles and muscle forces; however, its high inferring time makes it inappropriate for some applications. The RF model provided the best joint moments’ prediction results in the intra and inter-subject examinations. Based on the figures representing the models’ predictions for one gait cycle, the RF models provided smoother outputs, in addition to having lower prediction errors compared to other models. The RF algorithm is less prone to overfitting than other models^46^, which might explain its higher performance. Moreover, RF is a tree-based model and naturally ranks features by how well they improve the model’s performance and only uses the most important features to build trees. The good performance of the CNN models is due to their ability to automatically recognize relevant features, learn spatially correlated features, and create hierarchical representations of the input data. The lowest inferring time was related to the CNN model (0.23 s), making it suitable for real-time prediction by leveraging the power of parallel processing. Regardless of the ML model’s type, the level of prediction accuracy decreased (lower R^2^ and higher RMSE and MAE) for the inter-subject examination (when the training dataset did not include any of the testing subjects' trials). This can be partially explained by the fact that individuals' joint motion characteristics are distinct^51^. By including more participants' data for training the ML models, better predictions would be expected. Most outliers in Figs. 3 and 5 are related to two specific participants for whom the models provided poor estimations. This might be because of their particular walking patterns compared to other participants in inter-subject examination or walking unnaturally with diverse gait patterns in different trials in the intra-subject examination due to lab’s constraints.

To avoid the limitations of the traditional OMC systems, like the need for expensive equipment in a controlled environment and time-consuming data processing, previous studies have developed different algorithms to estimate joint kinematics from IMUs^51–62^. One of these algorithms used filtering approaches to cope with IMU sensor noise and integration drift^51,59–62^. While these algorithms succeeded in reproducing a similar joint angle waveform, the offset between IMU results and OMC systems is considered relatively high compared to our results. The RMSE ranging from $5^\circ$ to $10.14^\circ$ in the hip joint angle in the sagittal plane was previously reported^52,53,55–58,60,62^, while the present study achieved an RMSE of $1.38^\circ$ and $4.79^\circ$ for intra and inter-subject examinations, respectively. Our model produced lower RMSEs in knee joint flexion/extension ($1.85^\circ$ and $5.46^\circ$ for intra and inter-subject examinations, respectively), compared to other studies with reported RMSE between $4.1^\circ$ to $11.22^\circ$^52,53,55–58,62^. The accuracy of our model for ankle joint dorsi/plantarflexion angle prediction (2.14 $^\circ$ and $6.52^\circ$ for intra and inter-subject examinations) was comparable to previous studies with an RMSE of $1.9^\circ$ to $9.75^\circ$^52,53,55,57,62^. Other research groups achieved good accuracy by combining wearable sensors’ data with ML techniques for joint kinematics prediction^11–17,19^. The better performance of this approach (IMUs + ML model) provided low estimation errors in previous studies, especially in the intra-subject examinations with an RMSE ranging from $1.72^\circ$ to $3.58^\circ$ in hip flexion/extension^11,14,15^, from $2.21^\circ$ to $3.96^\circ$ in knee flexion/extension^11,12,14,15^ and from $1.81^\circ$ to $3.58^\circ$ in ankle dorsi/plantarflexion angle^11,12,14,15^. The performance of our RF model in the intra-subject examination was better than previous studies in the hip ($1.38^\circ$) and knee ($1.85^\circ$) and was in the range of these studies for ankle angle ($2.14^\circ$) in the sagittal plane. The higher prediction error for inter-subject examination is provided in some of the previous studies with hip flexion/extension angle RMSE ranging from $5.37^\circ$ to $8.85^\circ$^14,21,22^ (4.79$^\circ$ in the present study) and knee flexion/extension angle RMSE of $5.6^\circ$ to $7.41^\circ$^14,22^ ($5.46^\circ$ in the present study). However, the prediction error for the ankle dorsi/plantar flexion angle was lower in the previous studies with RMSE of $4.6^\circ$ to $5.5^\circ$^14,21,22^ ($6.53^\circ$ in the present study). In another study^19^, only the average RMSE of $7^\circ$ for all joint angles is reported, which is a higher prediction error compared to our results ($5.31^\circ$). Ren et al.^17^ developed five different ML models to predict hip, knee, and ankle joint angles in the sagittal plane and achieved MAE = $4.6^\circ$, $7.38^\circ$, and $4.74^\circ$, respectively, by using the RF model. They illustrated that the RF model outperforms other ML models (SVR, NN, multiple linear regression (MLR), and eXtreme gradient boosting (XGboost)) for joint kinematics prediction. The RF model in the current study carried out lower error than their model in hip and knee angles prediction by having MAE = $4.2^\circ$ and $4.59^\circ$, respectively, while we had higher errors than Ren et al.^17^ in predicting ankle dorsi/plantar flexion angle (MAE of $5.28^\circ$) for inter-subject examination. Long short-term memory neural network models were used in a recent study^20^ to estimate hip and knee joint angles in all planes of motion. The authors developed their models by using both measured and synthetic IMU data. When they used measured IMU data to train their models, RMSEs of $7.2^\circ$ for hip flexion/extension, $2.1^\circ$ for hip adduction/abduction, $4.2^\circ$ for hip rotation, and $2.9^\circ$ for knee flexion/extension angles were achieved. Their model outperformed our RF and CNN models in all of their targets except for hip flexion/extension ($4.79^\circ$ in our study). However, they didn’t perform any kind of cross validation to investigate the generalizability of their model. By changing training and testing datasets, different results may be found. Accurate results were achieved in another study^16^, in which 70 participants’ data were used to examine the model. They reported the MAE of 3.73, 5.41, and 3.58 for hip, knee, and ankle angles in the sagittal plane, respectively. However, we had more accurate estimation for knee flexion/extension angle (MAE = 4.59$^\circ$). While most of the previous studies concentrated on joint range of motion in the sagittal plane, our study additionally included the pelvis in all planes, hip int/ext rotation and abd/add and ankle inv/eversion.

Fewer studies were conducted to investigate the performance of different ML models for joint kinetics estimation^12,23–25^ compared to joint kinematics. All previous studies would focus on specific lower-limb joint kinetics (e.g. knee and ankle moments in the sagittal plane^12^, knee adduction/abduction moment^23^, medial and lateral knee contact forces^24^, knee flexion/extension and adduction/abduction moments^25^), while the present study investigated the prediction accuracy for the pelvis (in three planes of motion), hip (in three planes of motion), knee (in the sagittal plane) and ankle (in sagittal and frontal planes) joint moments during gait. The RF model presented in our study achieved an RMSE of 0.066 Nm/kg for ankle moment prediction in the intra-subject examination, which is more accurate compared to previous studies with an RMSE of 0.119 Nm/kg^12^. For the knee flexion/extension moment, we had lower accuracy in intra-subject examination compared to other studies (RMSE of 0.089 versus RMSE ranging from 0.042 to 0.068 Nm/kg^12,23^). However, our model outperformed another study in inter-subject examination (RMSE of 0.187 versus 0.27 Nm/kg)^25^. Higher prediction errors (BWBH%: Nm/bodyweight.bodyheight) compared to our results are reported^21^ for hip (1.78 BWBH%), knee (1.28 BWBH%), and ankle joint moments (1.39 BWBH%) in the sagittal plane. While we achieved %BWBH of 1.23 for the hip, 1.16 for the knee, and 1.08 for ankle joint moments.

To the best of our knowledge, there is no other study using wearable sensors’ data to estimate muscle forces. Ardestani et al.^63^ used an NN model to estimate muscle activations from EMG signals. They used muscle activations in a forward dynamic model to estimate lower-limb joint moments, but unfortunately, they didn’t report any prediction error for muscle activation or forces. In another study^64^, a Gaussian Mixture Regressor was employed to estimate muscle kinematics (fiber elongations and moment arms) and muscle activations from IMUs. They reported NRMSE lower than 30% of muscle activation for all muscles. In the present study, the lowest muscle forces prediction errors were associated with the biceps femoris long head muscle (NRMSE of 2.6% in intra and 4.5% inter-subject examinations). The highest NRMSEs were related to the semitendinosus muscle (NRMSE of 14.1%) in the intra-subject examination and biceps femoris short head muscle (NRMSE of 36.2%) in the inter-subject examination. A higher offset between actual and predicted values for muscle forces prediction compared to joint kinematics and kinetics can be seen in the figures depicting actual and predicted values. The lower accuracy in the estimation of muscle forces compared to other targets (joint kinematics and kinetics) was predictable. Data showed different muscle recruitment during walking between individuals and even between trials for the same participant, leading to less consistency in muscle forces across the population. Overall, compared to previous research, we predicted more targets at the same time with a multi-output RF model and achieved prediction errors within the range of what is reported in the literature.

Despite the number of participants (17 total), our RF model resulted in low prediction errors (comparable to the literature) in joints kinematics and kinetics estimation. We will investigate if increasing the number of participants to include a variety of gait profiles and self-selected speed provides more accurate estimations, specifically for muscle forces prediction. One limitation of the current study is the use of a multi-output RF model to predict many targets at the same time. A multi-output model helps us to improve the management of a high number of targets and allows us to decrease computational cost and monitor all intended targets simultaneously in real time. However, it may result in lower prediction accuracy by feeding many unrelated features to the model for some targets. The differences between the performance of a multi-output and a single-output model for the prediction of specific gait time series should be explored in a future study. Using separated single-output ML models would be more efficient in case we want to monitor a specific target.

The personalised musculoskeletal model for each participant was built using the gait 2392 OpenSim model, which lacks the degrees of freedom on the knee adduction/abduction, knee rotation, and ankle int/ext rotation, which does not allow us to study other planes of motion at the knee and ankle. The other limitation of this study was using an RF regressor to determine the rank of each feature. This can be a bias in favor of the RF model when comparing it with other ML models. However, we’ve shown that using selected features instead of all features is more efficient in the case of computational time, and its’ effect on the prediction accuracy of the CNN model is negligible. A further point to highlight is that we cannot guarantee that estimations using data from other labs will be as accurate as our own; this is largely due to differences in the equipment and sensors used. However, incorporating data from multiple labs into the training data set for our models can improve the models’ generalizability.

Although the findings of this study are very promising to benefit the community, more research is required to investigate the optimum number of IMUs needed to achieve these results. Looking back at the top features, it appears that some IMU data are not needed for 3D gait analysis. The optimal number and combination of IMUs can eliminate the need for seven sensors reducing data processing time and sensor cost. Reducing the number of sensors on the subjects’ bodies will also facilitate workflow implementation in the real world.

## Conclusion

This study showed that a combination of wearable sensors and ML techniques is an accurate and promising approach for improving traditional methods of gait time-series prediction. We also demonstrated that the higher performance of the RF and CNN models compared to other ML models make them more appropriate for predicting the lower limb’s joint kinematics, kinetics, and muscle forces, especially in the intra-subject prediction. Successful implementation of an intra-subject model enables us to remotely monitor changes in patients’ gait outside the clinic. While by having a precise inter-subject model, a gait analysis will be possible where an optical motion capture system is not available.

## Supplementary Information

Supplementary Information.

## Data Availability

The post-processed data (joint kinematics, joint kinetics, and muscle forces) along with the raw IMU and EMG data used in this study to build machine learning models are available on the open-source platform SimTK.org (https://simtk.org/projects/ml_sensors).
