# Supplementary material for: A comparison of machine learning models’ accuracy in predicting lower-limb joints’ kinematics, kinetics, and muscle forces from wearable sensors
Source: Sci Rep. 2023 Mar 28;13:5046. doi: 10.1038/s41598-023-31906-z (PMC10049990; doi:10.1038/s41598-023-31906-z)
Supplement: Supplementary file 1 — Supplementary Information. [file 41598_2023_31906_MOESM1_ESM.pdf]

Supplementary materials

Top features

Table 1 represents the top feature to predict each target. The name of each feature in Tsfresh is constructed by putting together three items. First, the name of the input component that is supplied to the feature extractor, second, the name of the feature extraction method available in tsfresh.feature_extraction.feature_calculators; and last, the name of the attribute that the feature corresponds to (only relevant for feature extractors that return more than one values). As can be seen, all top features for kinematics and kinetics prediction were extracted from IMU, and most top features for muscle predictions were extracted from EMGs.

Supplementary Table S1: Top feature to predict each target

Target	Top feature to predict the target
1. Pelvis tilt angle	RightFootIMU_accel.y__autocorrelation__lag_1
2. Pelvis obliquity angle	LeftThighIMU_gyro.z__cwt_coefficients__coeff_10__w_10__widths_(2, 5, 10, 20)
3. Pelvis rotation angle	LeftThighIMU _gyro.z__linear_trend__attr_"intercept"
4. Right Hip flex/ext. angle	RightThighIMU_gyro.z__agg_linear_trend__attr_"intercept"__chunk_len_10__f_agg_"max"
5. Right Hip add/abd. angle	RightThighIMU _gyro.z__cwt_coefficients__coeff_6__w_10__widths_(2, 5, 10, 20)
6. Right Hip rotation angle	RightThighIMU _gyro.y__fft_coefficient__attr_"real"__coeff_1
7. Right Knee flex/ext. angle	RightShankIMU_gyro.z__cwt_coefficients__coeff_5__w_20__widths_(2, 5, 10, 20)
8. Right Ankle dorsi/plantar angle	RightShankIMU _gyro.z__agg_linear_trend__attr_"intercept"__chunk_len_10__f_agg_"mean"
9. Right Ankle inv/eve angle	RightFootIMU_gyro.z__cwt_coefficients__coeff_7__w_10__widths_(2, 5, 10, 20)
10. Left Hip flex/ext. angle	RightFootIMU_gyro.y__index_mass_quantile__q_0.4
11. Left Hip add/abd. angle	LeftThighIMU_gyro.z__cwt_coefficients__coeff_2__w_20__widths_(2, 5, 10, 20)
12. Left Hip rotation angle	LeftThighIMU _gyro.z__fft_coefficient__attr_"angle"__coeff_1
13. Left Knee flex/ext. angle	LeftFootIMU_gyro.z__cwt_coefficients__coeff_9__w_20__widths_(2, 5, 10, 20)
14. Left Ankle dorsi/plantar angle	RightShankIMU_gyro.z__index_mass_quantile__q_0.1
15. Left Ankle inv/eve angle	PelvisIMU_accel.z__mean
16. Pelvis tilt moment	RightThighIMU _accel.x__cwt_coefficients__coeff_5__w_10__widths_(2, 5, 10, 20)
17. Pelvis obliquity moment	LeftThighIMU _accel.x__cwt_coefficients__coeff_2__w_5__widths_(2, 5, 10, 20)
18. Pelvis rotation moment	LeftThighIMU _gyro.z__cwt_coefficients__coeff_13__w_20__widths_(2, 5, 10, 20)
19. Right Hip flex/ext. moment	RightThighIMU _gyro.z__agg_linear_trend__attr_"intercept"__chunk_len_10__f_agg_"mean"
20. Right Hip add/abd. moment	RightThighIMU_gyro.z__cwt_coefficients__coeff_3__w_10__widths_(2, 5, 10, 20)
21. Right Hip rotation moment	RightFootIMU _gyro.y__agg_linear_trend__attr_"intercept"__chunk_len_10__f_agg_"mean"
22. Right Knee flex/ext. moment	RightShankIMU _gyro.z__cwt_coefficients__coeff_0__w_5__widths_(2, 5, 10, 20)

23. Right Ankle dorsi/plantar moment	RightThighIMU _accel.y__cwt_coefficients__coeff_14__w_20__widths_(2, 5, 10, 20)
24. Right Ankle inv/eve angle moment	RightThighIMU_gyro.z__cwt_coefficients__coeff_3__w_10__widths_(2, 5, 10, 20)
25. Left Hip flex/ext. moment	LeftThighIMU_gyro.z__agg_linear_trend__attr_"intercept"__chunk_len_50__f_agg_"mean"
26. Left Hip add/abd. moment	RightThighIMU_gyro.z__cwt_coefficients__coeff_4__w_10__widths_(2, 5, 10, 20)
27. Left Hip rotation moment	LeftThighIMU _gyro.z__cwt_coefficients__coeff_11__w_20__widths_(2, 5, 10, 20)
28. Left Knee flex/ext. moment	LeftShankIMU_gyro.z__cwt_coefficients__coeff_0__w_5__widths_(2, 5, 10, 20)
29. Left Ankle dorsi/plantar moment	LeftThighIMU_gyro.z__cwt_coefficients__coeff_0__w_10__widths_(2, 5, 10, 20)
30. Left Ankle inv/eve angle moment	RightFootIMU_accel.x__cwt_coefficients__coeff_3__w_20__widths_(2, 5, 10, 20)
31. Right Gluteus maximus	RightGastrocnemiusMedialis__abs_energy
32. Right Rectus femoris	RightRectusFemoris__mean_abs_change
33. Right Tibialis anterior	RightTibialisAnterior__time_reversal_asymmetry_statistic__lag_1
34. Right Vastus lateralis	RightVastusLateralis__linear_trend__attr_"stderr"
35. Right Semimembranosus	RightBicepsFemoris__change_quantiles__f_agg_"var"__isabs_False__qh_0.8__ql_0.2
36. Right Semitendinosus	RightSemitendinosus__root_mean_square
37. Right Gastrocnemius	RightShankIMU_accel.y__c3__lag_2
38. Right Soleus	RightThighIMU_gyro.z__cwt_coefficients__coeff_1__w_10__widths_(2, 5, 10, 20)
39. Right Biceps femoris-short head	RightBicepsFemoris__agg_autocorrelation__f_agg_"var"__maxlag_40
40. Right Biceps femoris-long head	RightShankIMU_gyro.z__cwt_coefficients__coeff_0__w_2__widths_(2, 5, 10, 20)
41. Left Gluteus maximus	LeftThighIMU_accel.x__cwt_coefficients__coeff_4__w_20__widths_(2, 5, 10, 20)
42. Left Rectus femoris	RightRectusFemoris__change_quantiles__f_agg_"mean"__isabs_True__qh_1.0__ql_0.4
43. Left Tibialis anterior	Left_TibialisAnterior__time_reversal_asymmetry_statistic__lag_1
44. Left Vastus lateralis	RightThighIMU_accel.x__linear_trend__attr_"slope"
45. Left Semimembranosus	LeftSemitendinosus__quantile__q_0.3
46. Left Semitendinosus	RightSemitendinosus__root_mean_square
47. Left Gastrocnemius	LeftGastrocnemiusMedialis__abs_energy
48. Left Soleus	RightSoleus__quantile__q_0.1
49. Left Biceps femoris-short head	LeftFootIMU_accel.x__agg_autocorrelation__f_agg_"var"__maxlag_40
50. Left Biceps femoris-long head	LeftFootIMU_gyro.y__cwt_coefficients__coeff_5__w_20__widths_(2, 5, 10, 20)

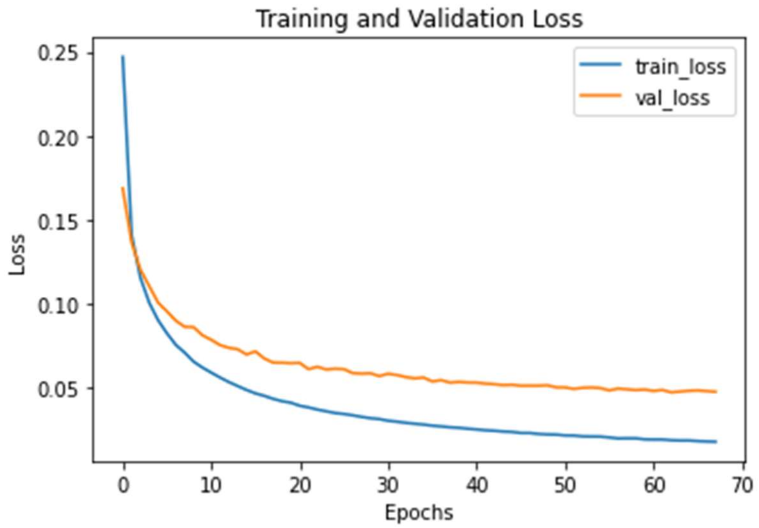

Supplementary Figure S1: Loss versus the number of epochs for the CNN model.

Supplementary Table S2: The training and testing time for all ML models using both selected features and all non-zero variance features.

		CNN	RF	SVM	MARS
**Using selected Features**	Training time (s) after selecting features	210.5	378.7	10020.9	14631.4
	Testing time (s)	0.23	0.44	945.38	1.041
**Using all non-zero variance features**	Training time (s)	1837.2	3561.7	825083.2	142914.7
	Testing time (s)	3.16	1.23	69580.15	2.81

Supplementary Table S3: Prediction accuracy (RMSE between ML models output and actual values) for all models using both selected features and all non-zero variance features.

	CNN RMSE			RF RMSE			SVM RMSE			MARS RMSE		
Targets / Used features	Kinematics (°)	Kinetics (Nm/kg)	Muscle forces (N)	Kinematics (°)	Kinetics (Nm/kg)	Muscle forces (N)	Kinematics (°)	Kinetics (Nm/kg)	Muscle forces (N)	Kinematics (°)	Kinetics (Nm/kg)	Muscle forces (N)
Selected features	6.93 ±3.4	0.23±0.112	75±81	6.4±2.5	0.203±0.09	67±79	6.77±2.6	0.20±0.093	65±65	7.91±3	0.273±0.14	99±112
All features	6.5+2.7	0.21±0.12	72±77	6.7±2.6	0.208±0.098	68±80	10.5±4.2	0.35±0.17	114±121	8.2±3.1	0.293±0.17	100±117

Joint kinematics

Supplementary Table S4: MAE ± std (°), and R2 (%) between OpenSim and ML models' outputs for kinematics predictions

	Intra-subject examination								Inter-subject examination							
	CNN		RF		SVM		MARS		CNN		RF		SVM		MARS	
	MAE (°)	R^2	MAE (°)	R^2	MAE (°)	R^2	MAE (°)	R^2	MAE (°)	R^2	MAE (°)	R^2	MAE (°)	R^2	MAE (°)	R^2
Pelvis tilt	0.81 ± 0.37	94	0.7 ± 0.31	96	0.8 ± 0.49	95	0.98 ± 0.74	89	3.68 ± 2.46	12	3.73 ± 3.12	17	3.83 ± 2.66	8	4.16 ± 2.58	-7
Pelvis obl.	0.84 ± 0.3	96	0.58 ± 0.16	98	0.69 ± 0.23	98	1.05 ± 0.72	93	3.43 ± 1.61	45	2.41 ± 0.9	72	3.1 ± 1.4	55	3.6 ± 2.32	52
Pelvis int/ext rot	1.61 ± 0.61	85	1.2 ± 0.28	92	1.32 ± 0.32	91	1.74 ± 1.0	76	3.12 ± 0.87	45	2.72 ± 0.88	57	3.33 ± 1.19	35	2.98 ± 0.82	52
Hip flex/ext	1.75 ± 1.02	97	1.09 ± 0.28	99	1.43 ± 0.56	99	1.83 ± 1.03	97	5.12 ± 2.89	82	4.2 ± 2.42	86	6.36 ± 4.55	69	5.22 ± 3.62	68
Hip add/abd	1.15 ± 0.43	96	0.78 ± 0.17	99	0.97 ± 0.28	98	1.28 ± 0.74	95	3.98 ± 1.54	61	3.14 ± 1.28	79	3.82 ± 1.75	66	4.38 ± 2.27	66
Hip int/ext rot	1.94 ± 0.61	92	1.54 ± 0.46	95	1.64 ± 0.48	95	2.12 ± 1.24	88	5.96 ± 1.88	23	6.0 ± 1.64	33	6.29 ± 2.03	18	6.48 ± 2.71	16
Knee flex/ext	2.26 ± 1.07	97	1.42 ± 0.27	99	1.77 ± 0.31	99	2.51 ± 0.92	97	5.69 ± 2.68	84	4.59 ± 1.87	87	6.74 ± 4.47	76	6.99 ± 4.66	64
Ankle d/p flex	2.13 ± 1.22	91	1.65 ± 1.13	95	1.97 ± 1.35	93	2.85 ± 2.42	83	5.93 ± 2.87	58	5.28 ± 2.51	61	6.14 ± 3.1	51	6.99 ± 3.96	49
Ankle inv/eve	2.41 ± 0.94	90	1.92 ± 0.68	93	2.04 ± 0.88	92	2.77 ± 1.18	85	6.37 ± 1.82	17	7.23 ± 2.74	28	6.47 ± 3.36	26	8.45 ± 2.85	4

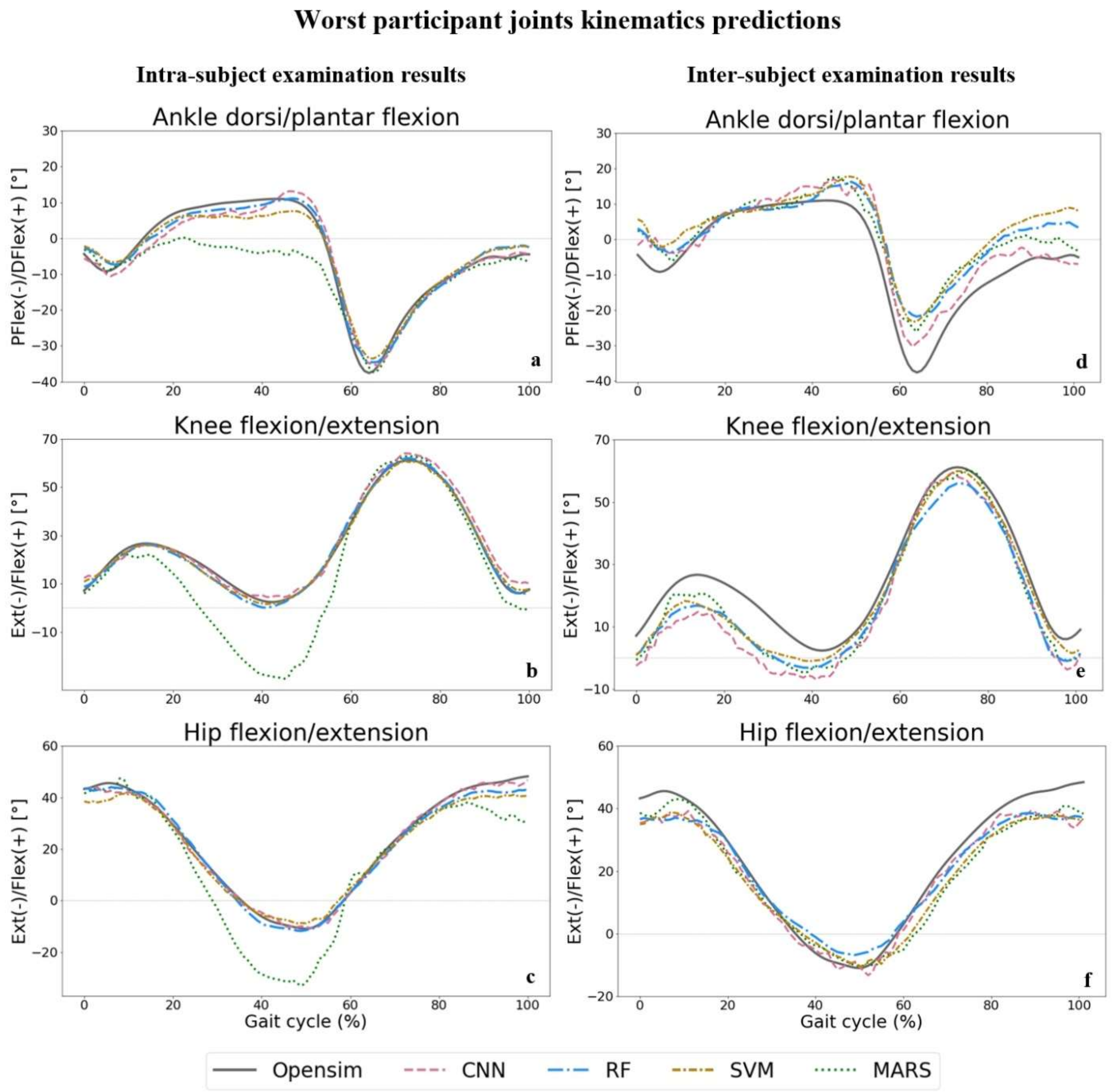

Supplementary Figure S2: Joint angles predictions by ML models compared to joint angles derived from OpenSim IK tool (solid grey line) across one gait cycle for ankle dorsi/plantar flexion (**a** for intra and **d** for inter-subject), knee flexion/extension (**b** for intra and **e** for inter-subject), and hip flexion/extension (**c** for intra and **f** for inter-subject) angles.

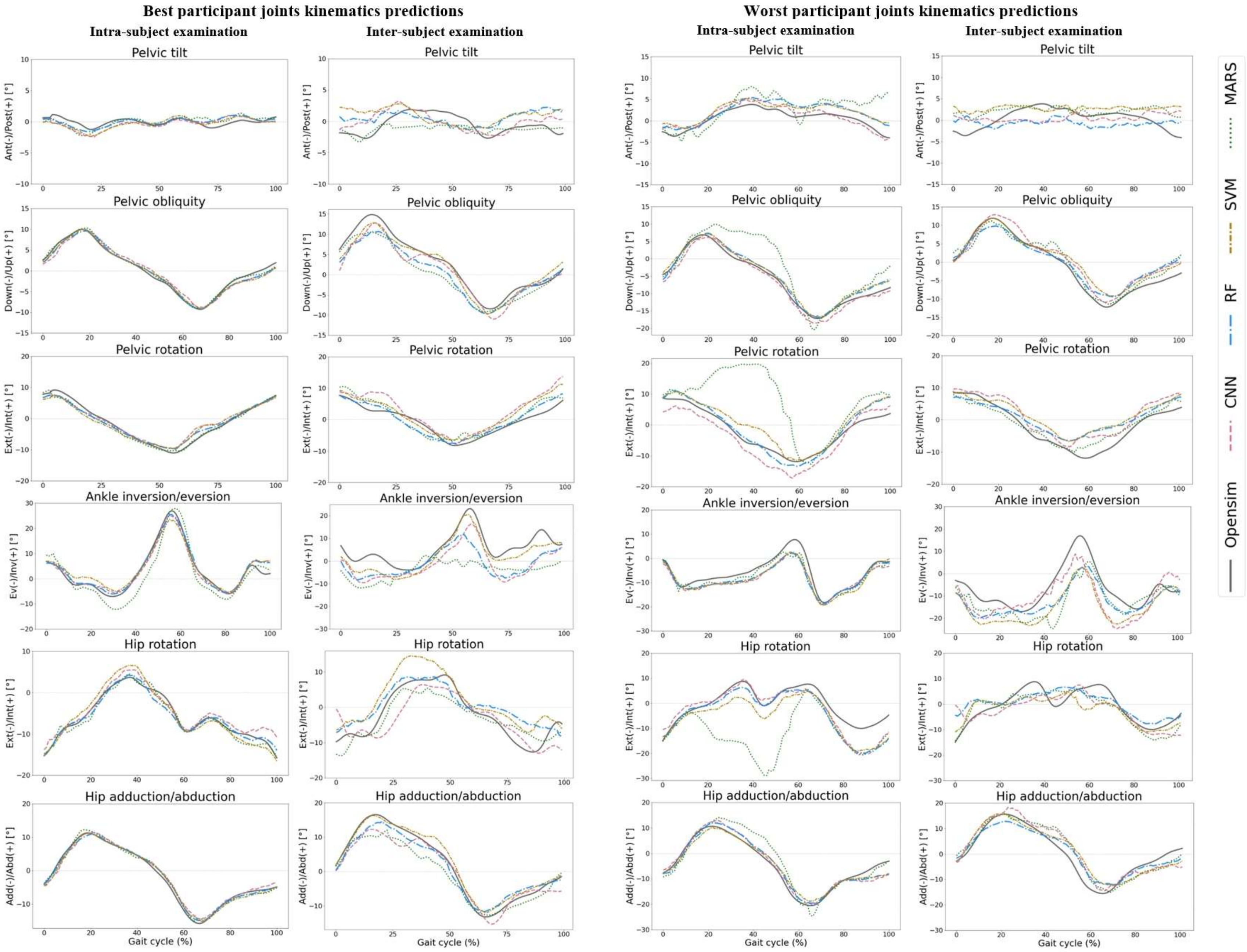

Supplementary Figure S3: Joint kinematics predictions by ML models compared to OpenSim IK output (solid grey line) across one gait cycle for Best and worst participants

Joint Kinetics

Supplementary Table S5: MAE ± std (Nm/kg), and R2 (%) between OpenSim and ML models' outputs for kinetics predictions

	Intra-subject examination								Inter-subject examination							
	CNN		RF		SVM		MARS		CNN		RF		SVM		MARS	
	MAE	R^2	MAE	R^2	MAE	R^2	MAE	R^2	MAE	R^2	MAE	R^2	MAE	R^2	MAE	R^2
Pelvis tilt	0.178 ± 0.05	48	0.136 ± 0.046	67	0.151 ± 0.046	56	0.207 ± 0.1	31	0.247 ± 0.05	17	0.201 ± 0.047	33	0.211 ± 0.046	22	0.276 ± 0.104	-3
Pelvis list	0.116 ± 0.038	60	0.091 ± 0.033	73	0.099 ± 0.039	66	0.133 ± 0.063	42	0.202 ± 0.044	18	0.154 ± 0.045	25	0.17 ± 0.048	12	0.176 ± 0.033	9
Pelvis rot.	0.046 ± 0.014	63	0.037 ± 0.013	74	0.042 ± 0.014	67	0.049 ± 0.017	56	0.077 ± 0.021	29	0.06 ± 0.022	36	0.065 ± 0.022	24	0.071 ± 0.03	31
Hip flex/ext	0.114 ± 0.033	85	0.078 ± 0.027	92	0.098 ± 0.028	88	0.131 ± 0.056	78	0.189 ± 0.051	60	0.14 ± 0.04	77	0.177 ± 0.078	62	0.181 ± 0.086	73
Hip add/abd	0.083 ± 0.021	93	0.059 ± 0.014	96	0.07 ± 0.015	95	0.097 ± 0.027	89	0.16 ± 0.038	72	0.12 ± 0.053	83	0.176 ± 0.099	62	0.172 ± 0.055	72
Hip rot.	0.023 ± 0.005	88	0.017 ± 0.003	93	0.019 ± 0.003	92	0.025 ± 0.005	86	0.046 ± 0.011	48	0.037 ± 0.012	67	0.041 ± 0.014	50	0.047 ± 0.014	57
Knee flex/ext	0.088 ± 0.029	89	0.061 ± 0.015	95	0.074 ± 0.017	92	0.095 ± 0.024	88	0.186 ± 0.078	49	0.137 ± 0.068	71	0.163 ± 0.094	53	0.175 ± 0.081	65
Ankle d/p flex	0.072 ± 0.029	96	0.039 ± 0.009	98	0.056 ± 0.012	97	0.069 ± 0.013	96	0.158 ± 0.087	74	0.117 ± 0.083	84	0.175 ± 0.128	69	0.187 ± 0.109	74
Ankle inv/eve	0.03 ± 0.007	87	0.02 ± 0.005	92	0.023 ± 0.005	91	0.03 ± 0.009	85	0.079 ± 0.028	27	0.05 ± 0.021	46	0.056 ± 0.023	39	0.076 ± 0.041	24

Worst participant joints kinetics predictions

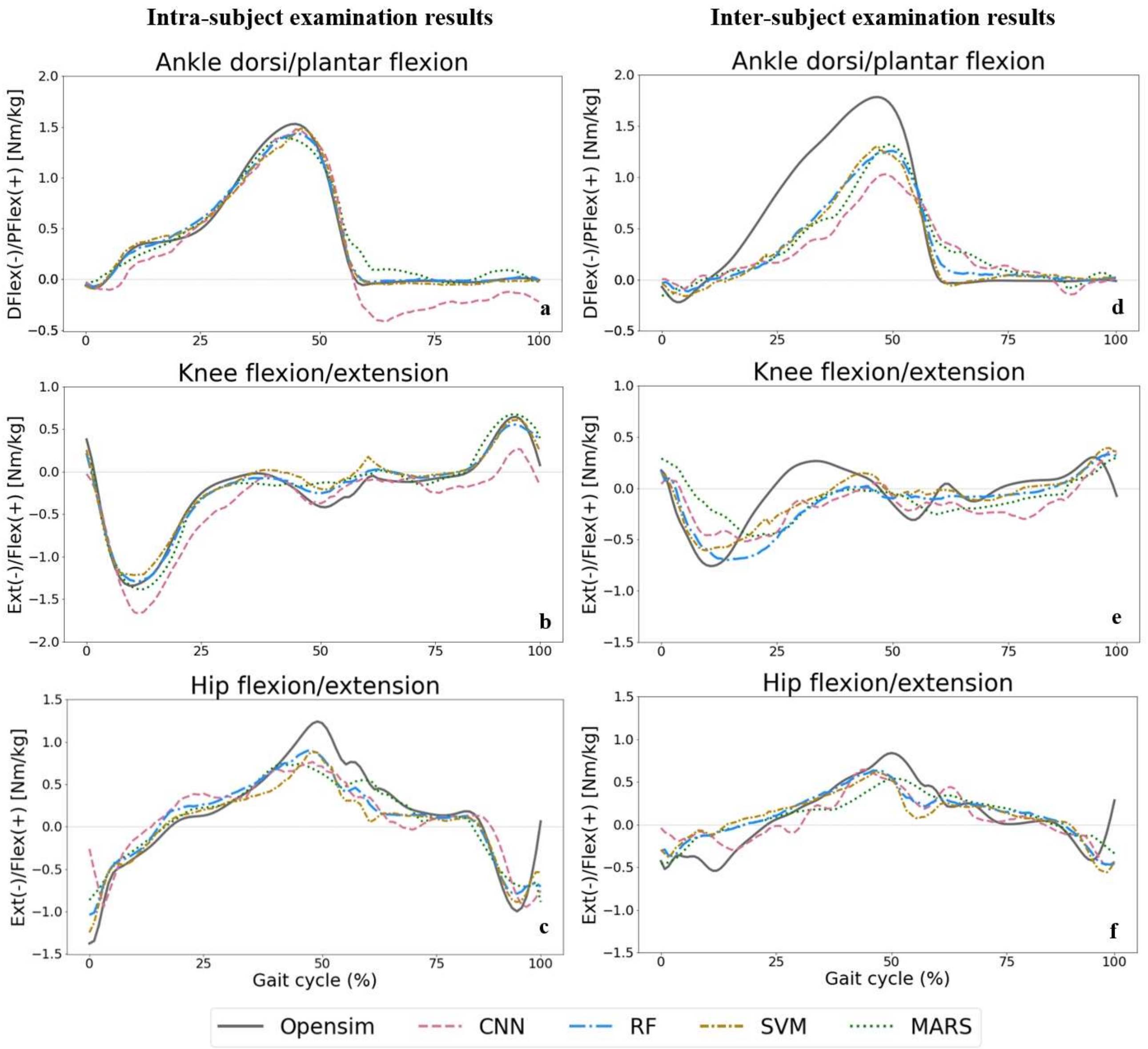

Supplementary Figure S4: Joint moments predictions by ML models compared to joint moments derived from OpenSim ID tool (solid grey line) across one gait cycle for ankle dorsi/plantar flexion (a for intra and d for inter-subject), knee flexion/extension (b for intra and e for inter-subject), and hip flexion/extension (c for intra and f for inter-subject) moments.

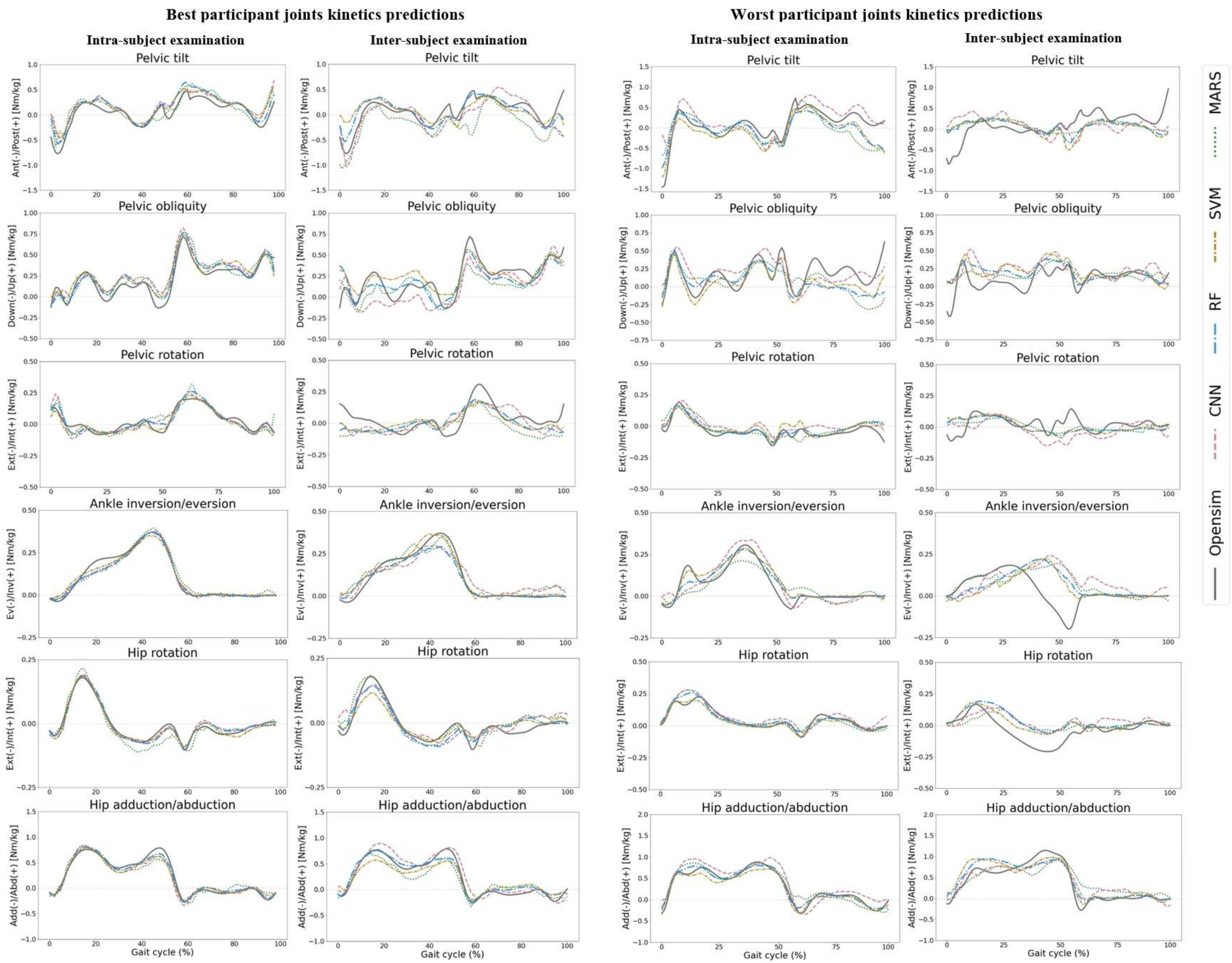

Supplementary Figure S5: Joint kinetics predictions by ML models compared to OpenSim ID output (solid grey line) across one gait cycle for Best and worst participants

Muscle forces

Supplementary Table S6: MAE ± std (N), and R2 (%) between CEINMS and ML models' outputs for muscle forces' predictions

	Intra-subject examination								Inter-subject examination							
	CNN		RF		SVM		MARS		CNN		RF		SVM		MARS	
	MAE	R^2	MAE	R^2	MAE	R^2	MAE	R^2	MAE	R^2	MAE	R^2	MAE	R^2	MAE	R2
Gluteus maximus	34.0 ± 18.0	67	20.0 ± 10.0	85	24.0 ± 12.0	84	33.0 ± 15.0	64	57.0 ± 17.0	26	42.0 ± 10.0	28	43.0 ± 13.0	22	73.0 ± 42.0	3
Rectus femoris	51.0 ± 25.0	74	35.0 ± 17.0	91	45.0 ± 22.0	88	53.0 ± 21.0	85	115.0 ± 69.0	20	96.0 ± 45.0	20	108.0 ± 52.0	-2	132.0 ± 64.0	1
Tibialis anterior	22.0 ± 11.0	80	16.0 ± 8.0	96	19.0 ± 9.0	95	25.0 ± 14.0	92	41.0 ± 13.0	25	44.0 ± 16.0	22	56.0 ± 22.0	-14	85.0 ± 39.0	-15
Vastus Lateralis	142.0 ± 89.0	80	91.0 ± 53.0	87	114.0 ± 62.0	84	126.0 ± 61.0	81	282.0 ± 104.0	26	206.0 ± 65.0	48	250.0 ± 128.0	28	337.0 ± 205.0	13
Semimem-branosus	18.0 ± 7.0	59	12.0 ± 4.0	73	16.0 ± 5.0	65	19.0 ± 7.0	44	32.0 ± 7.0	12	25.0 ± 5.0	30	26.0 ± 8.0	17	29.0 ± 8.0	17
Semiten-dinosus	26.0 ± 10.0	75	16.0 ± 7.0	82	20.0 ± 8.0	78	27.0 ± 12.0	67	45.0 ± 13.0	50	30.0 ± 6.0	62	34.0 ± 13.0	54	47.0 ± 13.0	40
Gastrocnemius	45.0 ± 32.0	89	32.0 ± 15.0	99	40.0 ± 16.0	99	55.0 ± 23.0	87	115.0 ± 45.0	40	177.0 ± 83.0	31	295.0 ± 141.0	-23	316.0 ± 169.0	-35
Soleus	105.0 ± 46.0	86	62.0 ± 31.0	91	79.0 ± 29.0	90	96.0 ± 46.0	73	222.0 ± 87.0	53	160.0 ± 53.0	65	192.0 ± 89.0	57	221.0 ± 93.0	49
Biceps femoris-sh	4.0 ± 3.0	70	3.0 ± 2.0	80	4.0 ± 2.0	74	4.0 ± 2.0	67	9.0 ± 4.0	28	7.0 ± 4.0	44	7.0 ± 5.0	-2	9.0 ± 4.0	-25
Biceps femoris-lh	18.0 ± 7.0	76	10.0 ± 4.0	85	14.0 ± 5.0	80	18.0 ± 7.0	72	31.0 ± 12.0	38	22.0 ± 7.0	56	23.0 ± 13.0	50	31.0 ± 13.0	38

Worst participant muscle forces predictions

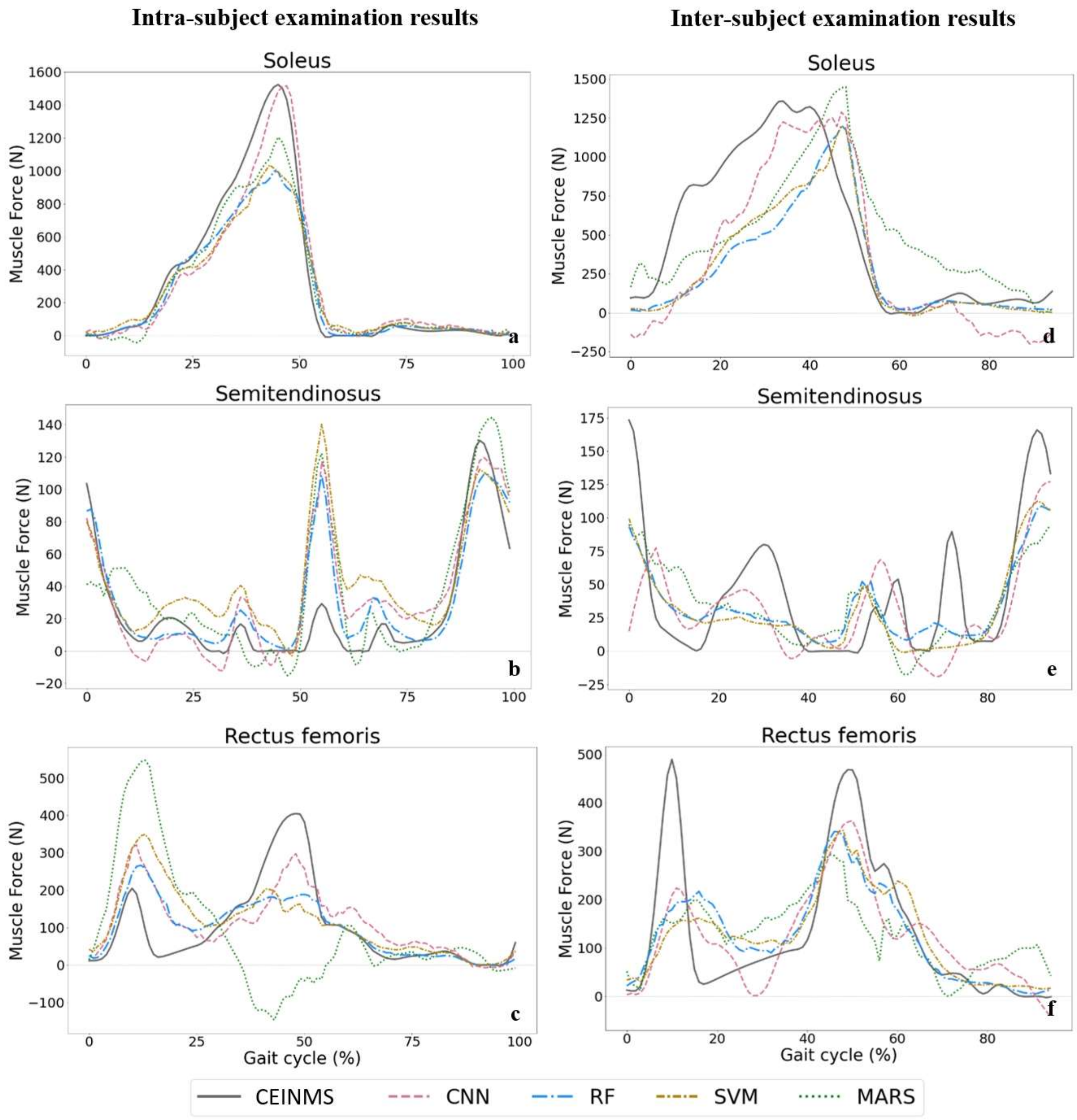

Supplementary Figure S6: Muscle forces predictions by ML models compared to CEINMS outputs (solid grey line) across one gait cycle for soleus (a for intra and d for inter-subject), semitendinosus (b for intra and e for inter-subject), and rectus femoris (c for intra and f for inter-subject).

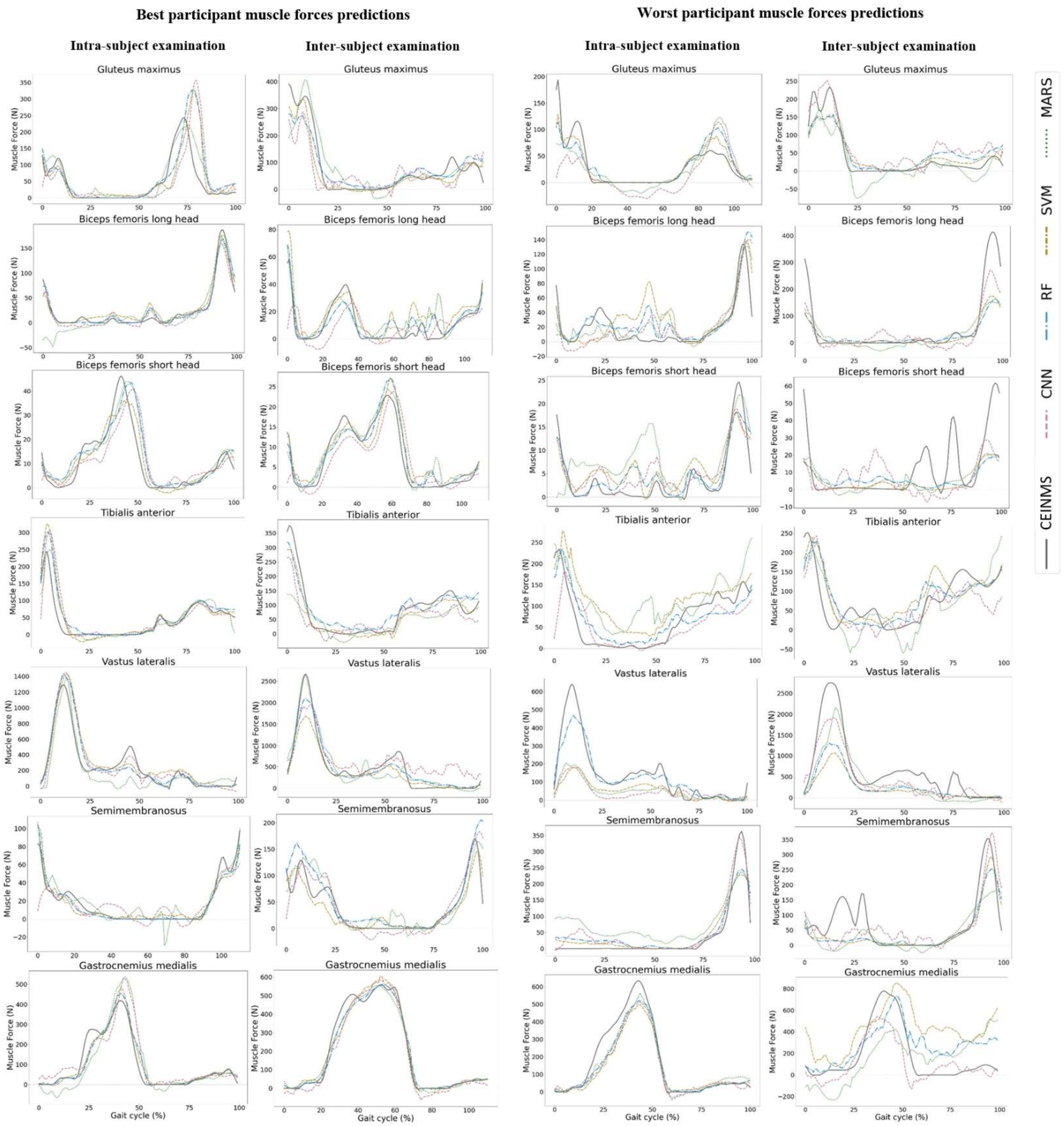

Supplementary Figure S7: Muscle forces predictions by ML models compared to CEINMS output (solid grey line) across one gait cycle for soleus, semitendinosus, and rectus femoris for Best and worst participants
